# Ultrasound-guided transversus abdominis plane block as an effective anesthetic technique for transverse colostomy in a high-risk elderly patient: A case report

**DOI:** 10.3389/fmed.2023.1102540

**Published:** 2023-03-13

**Authors:** Chao Li, Jingpu Shi, Huiqun Jia

**Affiliations:** Department of Anesthesiology, The Fourth Hospital of Hebei Medical University, Shijiazhuang, China

**Keywords:** transverse colostomy, transversus abdominal plane block, elderly, ultrasound, case report

## Abstract

Ultrasound-guided transversus abdominis plane (TAP) block is considered to be one of most prevalent and effective adjuvant analgesic methods for various abdominal surgeries. However, whether TAP blocks can be used alone as an effective anesthetic technique in minor abdominal operations has rarely been reported. Here we presented a 66-year-old male who had sustained right somatic dysfunction and mild brain dysfunction caused by cerebral infarctions and poorly treated hypertension. The patient received a confine operation of transverse colostomy to alleviate an intestinal obstruction caused by rectal cancer. A 22G needle was advanced in the plane under ultrasound guidance until it reached the TAP. A total of 10 mL 0.375% ropivacaine with 5 mg dexamethasone and 10 μg dexmedetomidine was injected into the TAP. The operation went stably and smoothly without any complaints. After the operation, the patient returned to the care of the surgical recovery staff with patient-controlled intravenous analgesia (PCIA) containing 0.7 mg/kg oxycodone and 2.5 μg/kg dexmedetomidine. During the perioperative period, the elderly patient did not experience apparent or unbearable pain. All these evidences indicated the ultrasound-guided subcostal and lateral TAP block was a simple and effective procedure for transverse colostomy in a high-risk elderly patient.

## Introduction

Transversus abdominis plane (TAP) block was first reported by professor Rafi in 2001, and it mainly involves an injection of local anesthetic solution into a plane between the internal oblique muscle and transversus abdominis muscle to block the spinal roots from T6 to L1 and alleviate pain in the abdominal wall (1). However, traditional landmark-guided TAP blocks *via* the triangle of Petit have been questioned about their accuracy and have limited application (2).

With the advancement and popularization of ultrasound visualization technology, TAP block technology has become easier and safer, rapidly becoming one of the most important assistant methods for postoperative analgesia in abdominal surgery. A large number of studies have reported that ultrasound-guided TAP blocks were able to alleviate stress responses, reduce the use of anesthetics during procedures and provide for postoperative pain in abdominal surgeries (3–5). Although ultrasound-guided TAP block is primarily used for postoperative analgesia management of various abdominal operations under spinal or general anesthesia, TAP alone may also a technically feasible alternative to some minor surgeries involving the abdominal wall. However, there is little literature about ultrasound-guided TAP blocks as a sole and effective anesthetic technique for transverse colostomy.

Here, we present a successful case in which ultrasound-guided TAP block provided surgical anesthesia for transverse colostomy in a high-risk elderly patient with various comorbidities, particularly cerebral infarctions.

## Case report

**Figure 1:**
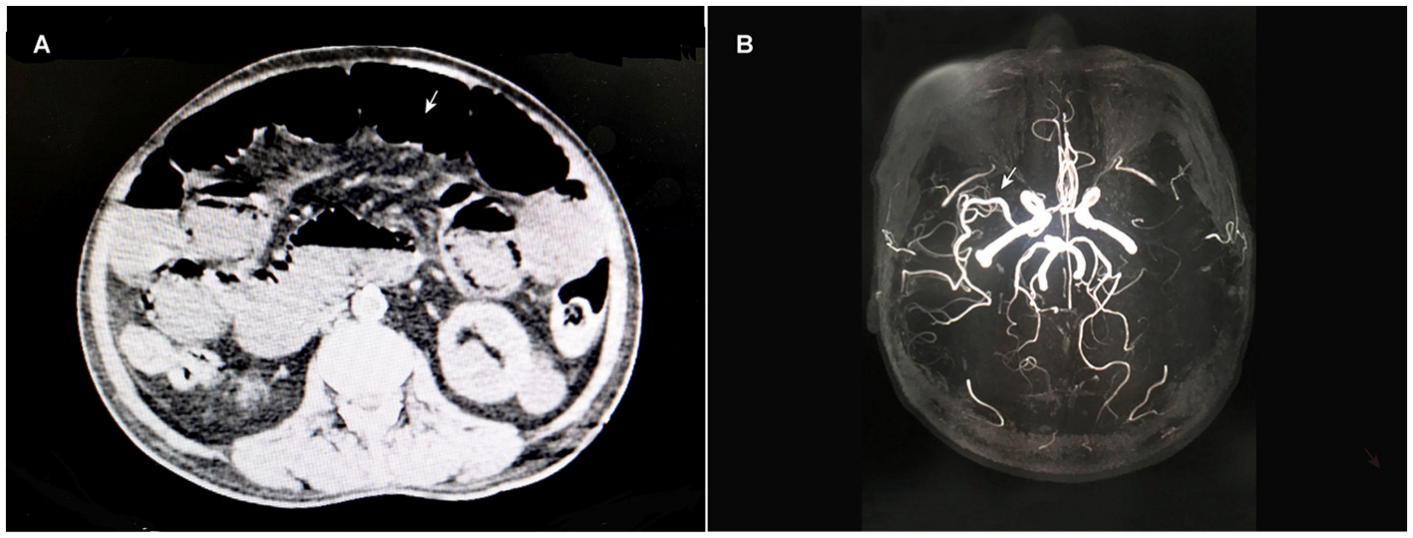
Abdominal CT scans **(A)** showed rectal cancer and an intestinal obstruction. The cerebral MRA image **(B)** showed that the left middle cerebral artery was invisible, while the right was normal.

A 66-year-old male patient (160 cm, 76 kg) was admitted to our hospital due to a change in stool frequency. He suffered from a severe bowel obstruction due to advanced rectum cancer diagnosed by pathological biopsy revealing poorly differentiated adenocarcinoma and plain computed tomography (CT), which is shown in Figure 1A.

He had a history of hypertension and cerebral infarction for 8 years and suffered from right somatic dysfunction and mild brain dysfunction. He irregularly used antihypertensive and anticoagulant drugs, including aspirin, clopidogrel, and nifedipine. During hospitalization, he was hemodynamically stable, and his blood pressure was maintained at approximately 140/90 mmHg. No obvious abnormalities were detected in routine blood investigations, including complete blood cell count, coagulation function and biochemical function tests. Electrocardiography (ECG) exhibited a normal sinus electrocardiogram, and echocardiography showed an ejection fraction of 67%, aortic valve degeneration and decreased left ventricular diastolic function. Chest CT findings displayed chronic inflammatory changes and multiple bullae in both lungs. The results of cranial magnetic resonance imaging (MRI) showed multiple cerebral infarctions and white matter degeneration, while cerebral vascular magnetic resonance angiography (MRA) was consistent with atherosclerosis, accompanied by left middle cerebral artery invisible, as shown in Figure 1B. All of these aspects of the patient's medical history and examination data indicated that he was a vulnerable elderly patient with multiple comorbidities.

After a multidisciplinary team discussion, the patient was assumed to have a high risk of traditional normal invasive surgery under general anesthesiology. Appropriate surgery and anesthesiology patterns were essential for the outcome and prognoses of his disease. Then, we chose transverse colostomy with ultrasound-guided TAP block as a safe and effective approach. After obtaining written informed consent for the ultrasound-guided TAP block, the patient was admitted to the operating room in a supine position. Then, the elderly patient received continuous oxygen inhalation through nasal catheter and routine monitoring includes ECG, continued non-invasive blood pressure and pulse oxygen saturation. An intravenous line was inserted, and a TAP block (subcostal + lateral approach) was performed bilaterally using an ultrasound machine with a linear probe (6).

**Figure 2:**
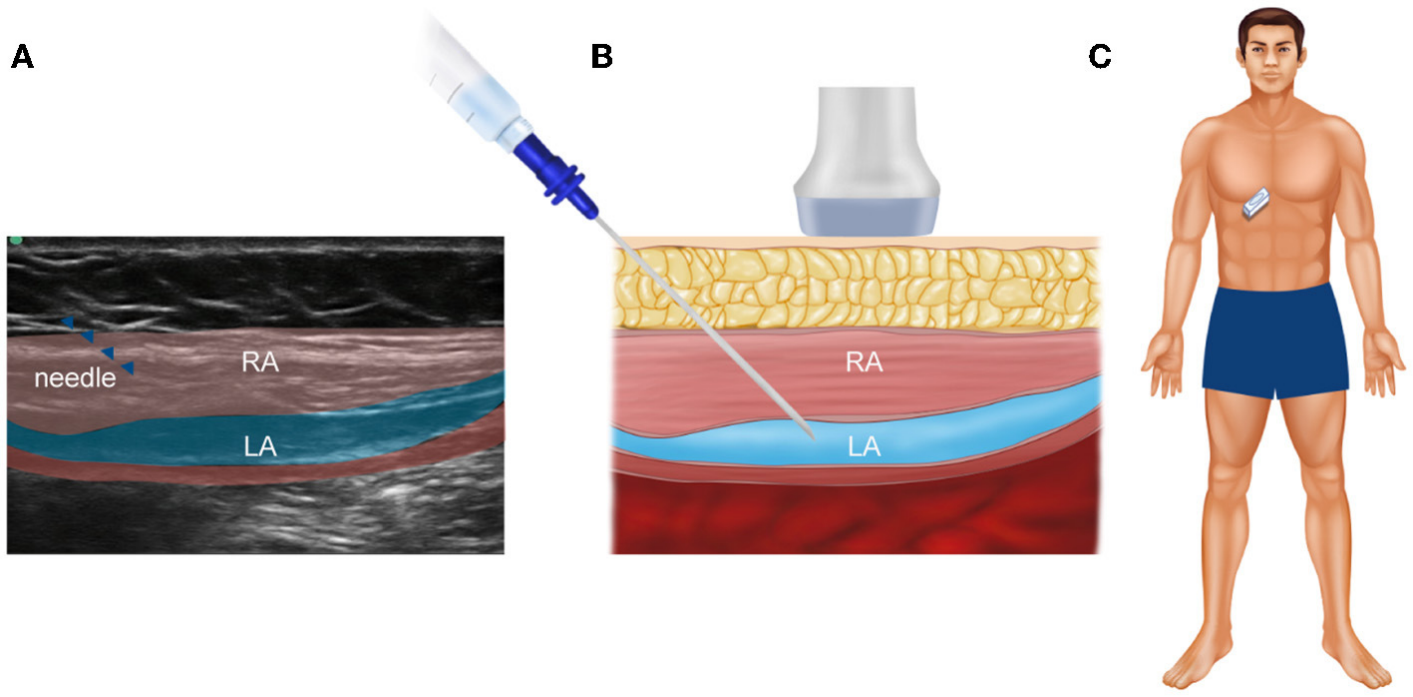
An ultrasound of the abdominal muscles and fascia in the subcostal TAP. **(A)** Real ultrasound image of the patient; **(B)** the corresponding schematic view, which is used to show the injection site more clearly; **(C)** the specific position of the ultrasonic probe on the patient's abdomen. RA, rectus abdominis; LA, local anesthetic.

**Figure 3:**
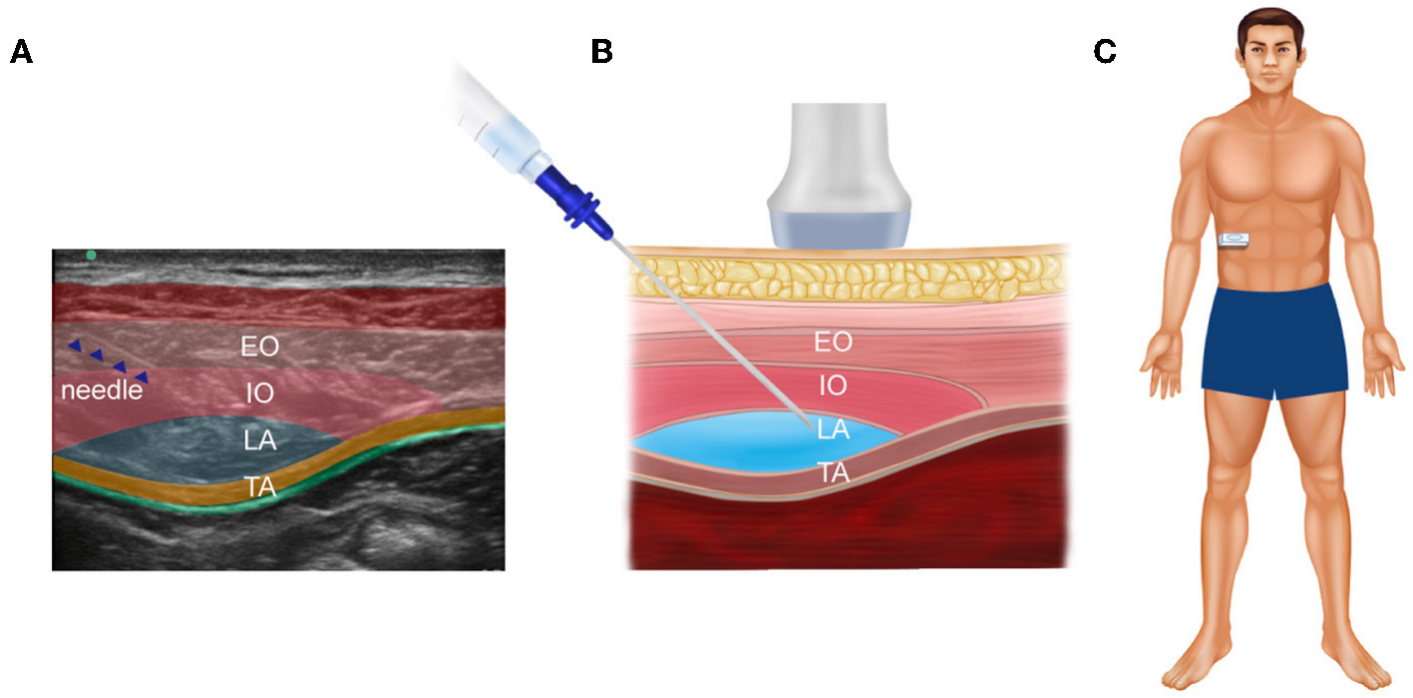
An ultrasound of abdominal muscles and fascia in the lateral TAP. **(A)** Real ultrasound image of the patient; **(B)** the corresponding schematic view, which is used to show the injection site more clearly; **(C)** the specific position of the ultrasonic probe on the patient's abdomen. EO, external oblique muscle; IO, internal oblique muscle; TA, transversus abdominis; LA, local anesthetic.

The patient's abdomen was cleaned with iodine and alcohol. The linear probe (HFL38x, 13-6 MHz transducer) of the Edge^®^ ultrasound system (Sonosite, Bothell, WA 98021, USA) was covered with a sterile protective cover. Local infiltration anesthesia was performed with 2 mL 2% lidocaine at the needle entry site. Then, a 22G needle (Stimuplex^®^ D, B. Braun, Melsungen, Germany) was advanced bilaterally in the plane under ultrasound guidance until it reached the TAP. A total of 10 mL 0.375% ropivacaine with 5 mg dexamethasone and 10 μg dexmedetomidine was injected at each site. Then, hypoechoic shades of local anesthesia spread were confirmed, as shown in Figures 2, 3.

The block area was checked with an acupuncture after 20 min following injection, and it was fully anesthetized. There were no complications for TAP block, including visceral damage, bleeding and local systemic toxicity. Then, surgeons were allowed to make a 5 cm vertical incision from the central abdomen to the peritoneal cavity. Before peritoneal manipulation, the patient had no complaints of discomfort or pain. When the intestinal and peripheral tissue adhesions were separated, the patient complained of abdominal discomfort. Hence, we administered 5 mg oxycodone intravenously. The operation lasted approximately for 60 min, and there were no surgical complications. The vital signs of the patient during the operation were relatively stable and smooth, and the patient remained pain free. After the operation, the patient returned to the surgical ward safely with a patient-controlled intravenous analgesia (PCIA) pump containing 0.7 mg/kg oxycodone and 2.5 μg/kg dexmedetomidine at a total volume of 100 mL. A visual analog scale (VAS) from 0 to 10 and observer's assessment of alertness/sedation (OAA/S) were used separately to evaluate the severity of pain and sedation during and 2 days after surgery. The duration of TAP block was approximate 10 h. The elderly patient did not complain of any pain and other adverse reactions including over sedation, respiratory depression, nausea, and vomiting occurred during and post operation. He was totally satisfied with this anesthesia method.

## Discussion

With an increasingly aging population worldwide, a growing number of elderly patients need to undergo various surgical interventions. However, elderly patients who require surgical operations often have multiple comorbidities that complicate their perioperative care and increase the risk of anesthesia (7). Adequate perioperative pain management reduces the severity of the surgery-induced stress response, leads to low morbidity rates and accelerates postoperative recovery (8). Previous studies have demonstrated the effectiveness of TAP blocks for postoperative pain control complementary to general anesthesia in various abdominal surgeries (9–11). However, few trials have used TAP blocks as a solo and effective technique in transverse colostomy surgery.

In this case, report, a fragile elderly man had irregularly treated hypertension and cerebral infarctions accompanied by right somatic dysfunction and mild brain dysfunction. Moreover, the chest CT and cerebral vascular MRA findings also confirmed additional complex and difficult comorbidities. Because of his comorbidities and medication history, local blocks, epidural blocks, and general anesthesia seemed inappropriate for this elderly man. Thus, we chose transverse colostomy with ultrasound-guided TAP block as a safe and effective approach for this unique patient with cerebral infarctions who suffered from an intestinal obstruction. As a result of this analgesic procedure, the patient achieved excellent analgesic effects during the perioperative period. Some factors may be related to achieving a good blocking effect. First, a multipoint injection in the ultrasound-guided TAP was able to comprehensively block the incisional pain. This is similar to previous reports that used bilateral continuous TAP blocks to achieve sufficient analgesia (12, 13). Second, one of ultrasound-guided TAP blocks' limitations is the short block duration, and reasonable selection of local anesthetic drugs with some adjuvant drugs are essential to prolong the duration of TAP block. Dexmedetomidine, dexamethasone, and clonidine are proved useful and suggested as effective adjuvants to prolong the duration (14). In this case report some dexmedetomidine and dexamethasone were added to ropivacaine to increase duration of the TAP block (approximate 10 h), which was slightly longer than duration of TAP blockade bupivacaine hydrochloride (5~8 h) in the previous research (15). Third, ultrasound-guided TAP block is a regional technique mainly for analgesia of the anterolateral abdominal wall, without effects on visceral pain (2). Oxycodone is effective to relieve visceral pain by mainly stimulating κ receptor. It was also found to be effective and efficient to regulate post-operative pain and inflammatory cytokine release in elderly patients undergoing laparoscopic gastrectomy (16). Hence, we added a small amount of oxycodone to alleviate abdominal visceral discomfort during the operation. Fourth, the PCIA pump with combination of dexmedetomidine and oxycodone provided effective analgesic effect, exert opioid-sparing effect and reduced the incidence of opioid related adverse reactions, such as nausea and vomiting, which is consistent with previous randomized controlled trial during open hepatectomy (17). Fifth, there was no TAP procedure related complications, such as visceral damage and bleeding, which may be attributed to skilled ultrasound-guided technology. Moreover, dexmedetomidine as an adjunct to ropivacaine in TAP block improved the block duration and efficiency, but did not significantly affect the sedation score, which was similar to the previous report (18).

In conclusion, ultrasound-guided TAP blocks can be considered an effective and attractive option for elderly and high-risk patients with several severe complex comorbidities, especially cerebral infarctions. This increase the interest in applying TAP blocks more widely in minor abdominal surgeries, chronic abdominal pain management and ICU patients requiring rescue analgesia. However, more research is needed to elucidate the usefulness and value of this anesthetic technology.

## Data availability statement

The raw data supporting the conclusions of this article will be made available by the authors, without undue reservation.

## Ethics statement

Ethical review and approval was not required for the study on human participants in accordance with the local legislation and institutional requirements. Written informed consent from the [patients/participants OR patients/participants legal guardian/next of kin] was not required to participate in this study in accordance with the national legislation and the institutional requirements. Written informed consent was obtained from the [individual(s) AND/OR minor(s)' legal guardian/next of kin] for the publication of any potentially identifiable images or data included in this article.

## Author contributions

CL and JS collected the data and wrote the paper. HJ reviewed and edited the manuscript. All authors read and approved the final manuscript.
